# Shatavari supplementation during eight weeks of resistance training increases training load, enhances skeletal muscle contractility and alters the skeletal muscle proteome in older women

**DOI:** 10.3389/fnut.2024.1498674

**Published:** 2025-01-06

**Authors:** Elsa Greed, Jack Pritchard, Lauren Struszczak, Esra Bozbaş, Georgia Ek, Jordan Acheson, Ben Winney, Aaliyah Qadir, Karl Ka-Lam Wong, Joanna Bowtell, Mary O’Leary

**Affiliations:** ^1^Faculty of Health and Life Sciences, Department of Public Health and Sport Sciences, University of Exeter, Exeter, United Kingdom; ^2^School of Sport, Exercise and Rehabilitation Sciences, University of Birmingham, Birmingham, United Kingdom; ^3^Department of Sport and Exercise Sciences, Institute of Sport, Faculty of Science and Engineering, Manchester Metropolitan University, Manchester, United Kingdom

**Keywords:** muscle, skeletal, exercise, resistance training, proteomics, aging

## Abstract

**Introduction:**

Shatavari is a herbal dietary supplement that may increase skeletal muscle strength in younger and older adults. Shatavari contains compounds with both estradiol-like and antioxidant properties, which could enhance muscle function. Postmenopausal women may derive the greatest benefit, as estrogen deficiency adversely impacts skeletal muscle function. However, mechanistic insights are limited and the effects of shatavari on muscle function require further characterization.

**Methods:**

In this randomized, double-blind trial, 17 young (23 ± 5 yr) and 22 older (63 ± 5 yr) women completed an 8-week leg resistance training programme. They consumed either a placebo or shatavari (1000 mg/d, equivalent to 26,500 mg/d fresh weight) supplement throughout. Pre and post training, measures of leg strength, neuromuscular function and vastus lateralis (VL) biopsies were obtained. Tandem-mass-tagged VL proteomic analyses were performed. Data were analyzed using a differential expression (Reactome) approach.

**Results:**

Shatavari supplementation increased 8-week training load in older women (leg press repetitions completed, *p* = 0.049, η_*p*_^2^ = 0.198; maximum weight lifted each week, *p* = 0.03, η_*p*_^2^ = 0.386; ANCOVA). There was no effect of shatavari on muscle strength post-training. VL half relaxation time was shortened post-training in older women supplemented with shatavari (post-training change: shatavari −11.74 ± 11.93%, placebo 0.42 ± 14.73%, *p* = 0.021; ANCOVA). Shatavari supplementation diminished the expression of extracellular matrix proteins in both cohorts. Expression of proteins related to striated muscle contraction, transcription and translation were decreased by shatavari supplementation in older women.

**Discussion:**

These novel observations support the notion that shatavari supplementation confers resistance to neuromuscular fatigue in older women. This could ameliorate sarcopenic declines in skeletal muscle function.

## 1 Introduction

Shatavari *(Asparagus Racemosus Willd*) is a herb that has been used in Ayurveda, for an array of purported therapeutic effects. Yet, empirical evidence of its efficacy remains limited. Notably, in the Ayurveda tradition, shatavari has been used to treat conditions related to the female reproductive system, due to its putative phytoestrogenic properties (1, 2). Shatavari is rich in phytoestrogens which are capable of binding to estradiol receptors (3, 4). Shatavari also contains many other bioactive compounds, including steroidal saponins, racAntioxidanemosides, racemosol, flavonoids and aspararagmine A (1, 5). Although limited, literature has suggested that shatavari has physiological effects in humans and recent research has suggested shatavari supplementation may increase skeletal muscle strength (2, 6, 7).

We have previously shown that 6 weeks of shatavari supplementation improved hand grip strength (HGS) in post-menopausal women. We also observed an increase in *vastus lateralis (VL)* myosin regulatory light chain phosphorylation; a known marker of effective myosin contractile function (7). Phosphorylation of Akt*^ser^*473 – an important signaling node in skeletal muscle hypertrophic responses – was also increased, but no alterations in phosphorylation status were found in other major effectors of muscle protein synthesis signaling. Subsequent analysis of the *VL* skeletal muscle proteome revealed shatavari-induced upregulation of proteins in pathways related to integrin/mitogen activated protein kinase signaling, and chemical synapse transmission (8). Integrins are important force sensors and central to instigating intracellular hypertrophic signaling responses to external forces. These observations suggest that shatavari supplementation may support muscle adaptations to resistance training.

One randomized controlled trial has shown beneficial effects of shatavari supplementation following an 8-week upper body strength training programme in a cohort of young males (6). Shatavari supplementation increased bench-press 1RM and repetitions until failure at 70% of one repetition maximum (1RM) following training. There was a greater rate of increase in training load observed in the shatavari group. Given that a decline in circulating estrogen after the menopause has been suggested to accelerate the age-related decline in skeletal muscle mass, we hypothesized that the phytoestrogen-rich shatavari may improve muscle strength in postmenopausal muscle (7, 9–11). However, the findings of Anders et al. in younger men suggest that other bioactives may also contribute to shatavari’s effects in skeletal muscle.

Indeed, compounds such as racAntioxidanemosides, racemosol, flavonoids and aspararagmine A have been suggested to have antioxidant effects (1, 12). Suboptimal redox balance in skeletal muscle is thought to alter skeletal muscle function (13). This has been particularly studied in the context of sarcopenia – the age-related decline in muscle mass and function. Reactive oxygen species (ROS) have been implicated in blunting protein synthetic responses and age-related anabolic resistance. Dietary supplements with antioxidant actions may counteract this, although evidence is limited (14, 15). In all age groups, myofibrillar calcium dynamics can be altered by ROS, in particular myofilament calcium sensitivity can be decreased by ROS (16). Dietary supplements with antioxidant actions may ameliorate this. The fatigue-resistance of shatavari-supplemented skeletal muscle described by Anders et al. supports this notion (6).

Further, it has been demonstrated that phytochemical-rich supplements can accelerate recovery from exercise-induced muscle damage (17, 18). We have recently demonstrated that 8 days of tart cherry supplementation accelerates such recovery and increased endogenous skeletal muscle antioxidant enzyme expression, including protein-level expression of glutathione peroxidase 3 (19). Enhanced endogenous antioxidant capacity could also explain the effects of shatavari on skeletal muscle.

The study of Anders et al. remains the only investigation of the effect of shatavari on skeletal muscle function during and after a resistance training intervention (6). This study was restricted to men and did not offer any insights to suggest a mechanism of shatavari action. Resistance training remains the gold-standard intervention for the amelioration of sarcopenia (20, 21). Such training is challenging to implement and does not completely reverse sarcopenic changes. Therefore, low-burden dietary adjuncts that increase the effectiveness of such training are desirable. Such dietary supplements may also be of interest in younger populations, where athletic performance or preventative health benefits may be desired. Given the prominence of phytoestrogens amongst the complex bioactive shatavari blend, we chose to conduct an 8-week leg resistance training study in young and older women. This double-blind placebo-controlled parallel groups trial aimed to determine the effect of shatavari supplementation on post-training 1RM (knee extension, leg press), NMF and the *VL* proteome. We hypothesized that shatavari would increase post-training knee extension 1RM (primary outcome) compared to a placebo control, with greater efficacy in the estrogen deficient post-menopausal women.

## 2 Materials and methods

### 2.1 Study overview

Two double-blind, placebo-controlled studies of a herbal supplement were approved by the University of Exeter’s Sport and Health Sciences Research Ethics Committee (Refs: v140922, 21-10-20-B-06). Young (18–35 yr) and older (≥55 yr) women were recruited through posters, Facebook ads and the local University of the Third Age chapter. Exclusion criteria included: <1-year post-menopause (older women only), self-reported or diagnosed menstrual irregularities (amenorrhea, anovulation, oligomenorrhea; young women only), undertaking >two sessions per week of lower limb resistance training, lower limb musculoskeletal injury within six months, recent non-steroidal anti-inflammatory drug or aspirin use, lidocaine allergy and any medical condition that would prevent resistance training. All participants completed a Physical Activity Readiness Questionnaire (PARQ) before engaging in physical activity. Twenty-six post-menopausal women and 18 younger women were recruited and randomized. Twenty-two older women (62.7 ± 5.3 yr; BMI 24.7 ± 3.9 kg.m^–2^; shatavari group (SH) *n* = 11, placebo group (PL) *n* = 11) and 17 younger women (22.9 ± 5.1 yr; BMI 24.0 ± 3.0 kg.m^–2^; SH *n* = 9, PL *n* = 8) completed the intervention. Four post-menopausal women withdrew from the intervention for medical reasons unrelated to the trial, and one younger woman withdrew from the study due to time constraints. Self-reported medication usage was recorded (Supplementary Table 1). A 2-week washout period was provided for participants consuming supplements exceeding the NHS recommended daily dosage.

**FIGURE 1:**
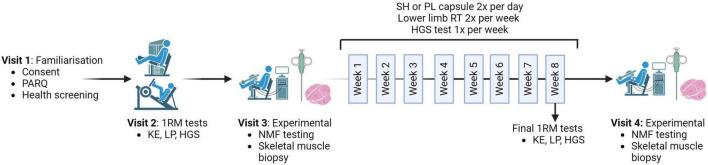
Schematic representation of the study protocol. The protocol begins with the one repetition maximum (1RM) visit, where strength measures of knee extension (KE), leg press (LP) and hand grip strength (HGS) are taken before the pre-measures visit and repeated in the final session of the training program. Both the pre- and post-visits include neuromuscular function (NMF) testing followed by a skeletal muscle biopsy. Participants received either 1000 mg/day of Shatavari (SH) or a Placebo (PL) for 8 weeks. The training program comprised two sessions per week (W), focusing on KE and LP. HGS was measured once per week. Created with BioRender.com.

The study protocol (Figure 1) included four main visits conducted alongside an 8-week training program at St Luke’s campus, University of Exeter. During Visit one, participants completed a health screening questionnaire, provided informed consent, and had their height and weight measured. Participants were familiarized with HGS and NMF testing protocols. Equipment settings were recorded for future visits. Prior to all subsequent non-training visits, participants arrived fasted and abstained from caffeine, alcohol, and strenuous exercise for 48 h.

At visit two, following a standardized warm-up, leg strength was assessed by determining 1RM for knee extension (KE) and leg press (LP) exercises on leg extension (Life Fitness, hammer strength select leg extension, SL20) and leg press (Body-Solid compact leg press, #GCLP100, Forest Park, IL, USA) machines with weight increasing with 3 min rest intervals until failure. Equipment settings were adjusted to ensure proper form. HGS was measured between 1RM KE and LP testing using a handgrip dynamometer (Takei 5001 Grip Dynamometer Analog, Takai Scientific Instruments Co., Ltd., Niigata City, Japan), following a standing version of the Southampton protocol (22). Three trials were conducted on each hand and the test hand was alternated. The best score for either hand was used for data analysis.

Prior to Visit three (pre-supplementation), participants recorded a 3-day food diary. NMF testing, blood sampling and *vastus lateralis (VL)* skeletal muscle biopsy were performed respectively. Prior to Visit four (post-supplementation), participants replicated their 3-day food diary intake. At Visit four, participants repeated Visit three procedures.

### 2.2 Neuromuscular function testing

Electromyography (EMG) was used to measure electrical activity during voluntary contractions and involuntary contractions evoked by peripheral nerve stimulation (PNS) in the *VL* and biceps femoris *(BF)*. Three adhesive electrodes (2 cm × 2 cm biotab, Boots UK Ltd., Nottingham, England) were placed on the participant’s dominant leg. The cathode electrode was placed 2 cm lateral to femoral artery in contact with the participant’s skin. The anode electrode was positioned on the gluteal fold directly posterior to the cathode. The final electrode was placed on the patella to reduce background noise up to 5Hhz. EMG electrodes (99.9% Ag, 10-mm length, 1 mm width, 10 mm pole spacing, CMRR > 80 dB, model DE2.1, DelSys Inc., Boston, MA, USA) were located in accordance with SENIAM guidelines and in alignment with the muscles fibers on the participants dominant leg on the *VL* and *BF*^1^ and fixed using double-sided adhesive and hypoallergenic medical tape. Electrodes connected to a high-voltage direct electrical current stimulator (Digitimer DS7AH Current Stimulator, Digitimer, Hertfordshire, UK), creating a single-pulse electrical stimulation (200-μs pulse duration) at the femoral nerve. Participants were positioned on a leg-extension dynamometer with their non-dominant leg secured in place. The dynamometer was connected to an in-line force transducer (Omni instruments, DBBE-150kg-003-OMN01), facilitating the measurement of force (N) during a kicking motion. A standard M-wave recruitment curve was performed to identify the maximum Mwave (23). The electrical stimulation was increased in 20 mA increments until the Mwave amplitude reached a plateau (Mmax). To confirm the maximal *VL* Mwave, an electrical current of 130% Mmax was applied. If Mwave increased, the recruitment curve was continued until a plateau was reached. Once Mwave plateau was achieved, five resting stimulations at 130% Mmax were performed, separated by 1 min. Following this, five non-stimulated and five stimulated maximal voluntary contractions (MVC) were performed, with alternation between stimulated and unstimulated attempts, with 1 min rest between them. During stimulated MVCs, a PNS pulse was introduced 1 s into the MVC (superimposed), followed by a second pulse 2 s after relaxation (potentiated twitch), both at a current equivalent to the 130% Mmax.

Data collection and analysis were conducted using Spike2 version 7 (CED, Cambridge, UK). The EMG data were recorded at a frequency of 2000Hz via an A/D converter (CED 1401power, Cambridge, UK), while force outputs were recorded at 200Hz through the force transducer and the converter. EMG signals from the *VL* were processed using a custom-written script to measure peak-to-peak Mwave amplitude and area, peak twitch (PT), contraction time (CT) and half relaxation time (HRT).

### 2.3 *Vastus lateralis* biopsy

A biopsy site was prepared on the lateral aspect of the dominant leg, overlying the *VL*. Providone-iodine 10% w/w cutaneous solution was used to sterilize the area. The skin and fascia were anesthetized with 2% lidocaine. A ∼ 0.8cm incision was created. A skeletal muscle sample ∼ 150 mg was obtained via the suction modified Bergstrom method (24). Muscle tissue samples were immediately frozen in liquid nitrogen or liquid nitrogen cooled isopentane and stored at –80°C until ready for analysis.

### 2.4 Supplementation and randomization

Following visit three, participants were randomized to one of two supplementation groups (1:1 allocation to SH or PL) using an online randomization generator. An independent researcher completed the randomization. Participants consumed two shatavari capsules daily (500 mg shatavari powder per capsule), equivalent to 26,500 mg fresh-weight shatavari root per day. Placebo capsules contained 500 mg magnesium stearate. Supplementation coincided with an 8-week resistance exercise program. Capsules were indistinguishable in appearance, smell and taste. Compliance was measured by counting the remaining capsules post-intervention.

### 2.5 Lower limb resistance training

The 8-week training programme consisted of two sessions per week of both LP and KE exercise. HGS was measured during the first session of each week between LP and KE exercises. Each session began with a two-set KE warm-up of 8 repetitions at 50% of the previous session’s final weight, followed by 3 repetitions at 70% of that weight, separated by a 2 min rest interval. Participants then performed exhaustive sets of KE and LP respectively. Training sessions progressed from 2 sets of 12–15 repetitions in weeks 1–3, to 3 sets of 10–12 in weeks 4–6 and 4 sets of 8–10 repetitions in weeks 7–8. The starting weight for participants’ initial training session was 70% 1RM. The weight was adjusted if participants had ease/trouble completing the 12–15 repetitions for the first week. The weight lifted was increased throughout the training programme when participants achieved two or more repetitions above the desired range for two consecutive sets. KE was increased in 3.5 kg increments and the LP in 2.5 kg increments. Throughout each set, participants received consistent verbal encouragement and feedback on technique and ranges of motion. To characterize training load, the maximum weight lifted (MWL) during training each week [calculated using the formula: MWL during training week (kg)/baseline 1RM (kg)] and the number of repetitions completed in each training week were calculated. During the final training visit in week eight, participants completed 1RM and HGS as per visit two.

### 2.6 Skeletal muscle fiber size quantification

*VL* muscle samples were aligned for transverse sections and sectioned on cryostat at −22°C at a set thickness of 10 μm. The cryosections were collected on glass slides, air dried and frozen in slide mailers at −80°C until staining. Cryosections were fixed in ice-cold acetone for 10 min. Cryosections were encircled with a hydrophobic barrier and washed once with phosphate buffered saline (PBS). A blocking solution was applied to the cryosections (5% v/v goat serum) for 1 hr at room temperature. Cryosections were washed briefly in PBS. Wheat germ agglutinin (Thermo Fisher, Alexa Fluor Plus 405 conjugate) was applied at a concentration of 2.5 μL/mL in PBS for 1 h. Cryosections were washed three times with PBS and kept at room temperature until dry. A drop of mountant (ProLong Diamond Antifade, Thermo Fisher) was added to each section and a coverslip applied. Cryosections were stored at 4°C until immunofluorescent imaging at 20 x magnification (Leica Thunder Dmi8 widefield microscope, Leica Microsystems). Fiber size was quantified using Open-CSAM (particle size 200 – infinity, circularity 0.2–1.0; incorrectly identified fibers were manually deleted or added following automated analysis) (25). Cross-sectional fibers were counted from three cryosections per *VL* sample, with sections being taken from portions of the sample at least 20 μm apart. The mean number of fibers counted per sample was 2382 (SD = 1364, minimum = 266, maximum = 2514).

### 2.7 Proteomic analysis

Skeletal muscle samples (∼15 mg) from 28 participants (older SH *n* = 8, PL *n* = 8; younger SH *n* = 5, PL *n* = 7) were subjected to proteomics analysis Samples were prepared in radioimmunoprecipitation assay (RIPA) buffer as previously described (8). Tandem mass tagged (TMT) labeling and high pH reversed-phase chromatography was performed at the University of Bristol, also as previously described (8). Samples were analyzed in four 15Plex experiments, each containing 14 experimental samples (7 ‘pre and post’ pairs) plus a common ‘reference’ sample.

The raw data files were processed and quantified using Proteome Discoverer software v2.4 (Thermo Fisher Scientific) and searched against the UniProt Human database (downloaded January 2022: 178486 entries) using the SEQUEST HT algorithm. Peptide precursor mass tolerance was set at 10 ppm, and MS/MS tolerance was set at 0.6 Da. Search criteria included oxidation of methionine (+ 15.995 Da), acetylation of the protein N-terminus (+ 42.011 Da) and Methionine loss plus acetylation of the protein N-terminus (−89.03 Da) as variable modifications and carbamidomethylation of cysteine (+ 57.0214) and the addition of the TMTpro mass tag (+ 304.207) to peptide N-termini and lysine as fixed modifications. Searches were performed with full tryptic digestion and a maximum of 2 missed cleavages were allowed. The reverse database search option was enabled, and all data was filtered to satisfy false discovery rate (FDR) of 5%.

Data were analyzed at the University of Exeter. Data were normalized to the total peptide amount in each sample and scaled using a pooled ‘reference’ sample common to both TMT experiments to facilitate the comparison of protein levels between experiments. Data were log2 transformed prior to analysis. Data were filtered to include only proteins that were detected in all samples (older 2913 proteins; younger 2915 proteins). Reactome (Reactome V8)^2^ was used to perform differential expression analyses. To account for skewed analysis that may appear due to inter-protein correlations, Reactome’s CAMERA algorithm estimates inter-protein correlations directly from the data to modify the test statistic for protein sets. Analyses were conducted on data from the younger and older cohorts separately. The purpose of these analyses was to gain additional molecular insights into the mechanisms of shatavari action via comparison with an age-matched placebo supplemented group. To compare the proteomes of the young and older cohorts statistically and comprehensively constitutes a different study and is outside the scope of this manuscript. The FDR (Benjamini-Hochberg-adjusted *p*-value < 0.05) and log2 fold change (Log2 FC) values are reported for both individual proteins and pathways. Reactome consists of thematic pathway ‘nodes’. For each node where an element reached FDR < 0.05, parent pathways with an FDR < 0.05 or the highest-level child pathway with an FDR < 0.05 are presented. Protein networks were visualized using in Cytoscape v3.10.0 using stringApp v2.0.1 (26). The full STRING network was used with a high confidence threshold of 0.7. Log2 FC values (shatavari vs placebo) were used to style the network, indicating expression of individual proteins. Network clustering was performed using the Markov clustering implementation in the clusterMaker2 Cytoscape app; the inflation value was set to 4.0.

### 2.8 Sample size

The sample sizes were based on the best available but extremely limited data in the literature: 1RM bench press data presented by Anders et al. (6). It was determined that 16 participants per group were required to detect a similarly large change in KE 1RM (*d* = 1.03, α = 0.05, 1-β = 0.08). Therefore, a sample size of *N* = 32 was planned for each study. However, given the lack of precedent surrounding these calculations and interim blinded analysis of KE data were planned once data from *N* = 22 participants had been collected. Proteomic analysis of *VL* samples was planned for a sub-group of participants. Hypothesis-free pathway level (vs individual protein) analyses are not readily conducive to traditional sample size calculations. Published and unpublished data from our group suggested that we would detect meaningful changes in the tissue-level (skeletal muscle) proteome with 8 participants per group (8).

### 2.9 Statistical analysis

One-way repeated ANCOVAs were conducted to determine the difference in NMF between placebo and shatavari groups in both young and older participants separately, controlling for baseline values. Bonferroni *post hoc* tests were used for multiple comparisons. The statistical analyses were performed in SPSS version 23 (SPSS Inc., Chicago, IL, USA). Statistical significance was set at *P* < 0.05 and data are presented as means ± SD. Where intuitive means ± SD cannot succinctly be presented in text, e.g., training load data, partial eta squared values (η_*p*_^2^) are reported.

## 3 Results

### 3.1 Adherence, compliance, and adverse events

Older women were fully compliant with their supplementation regime throughout the 8-week training period. Compliance in younger women was 97 ± 3%. Participants reported no serious adverse events during the study. One participant missed four out of 16 training sessions over the 8-week period. The mean training compliance was 93 ± 7% in younger women and 96 ± 6% in older women.

### 3.2 Training load, muscle strength and fiber size

**FIGURE 2:**
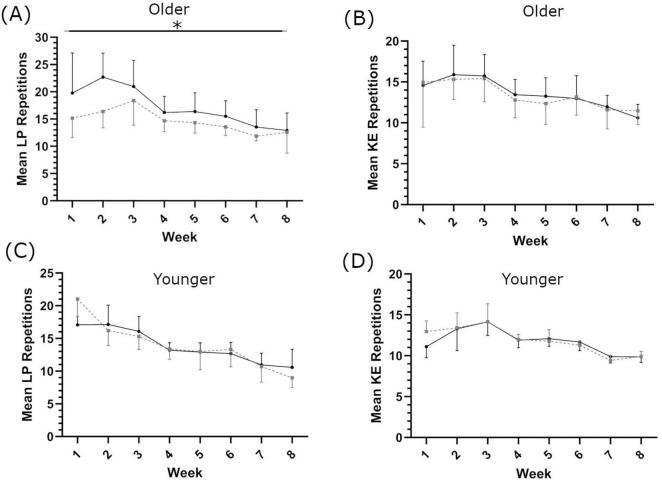
Mean repetitions completed each week for leg press [LP; **(A,C)**] and knee extension [KE; **(B**,**D)**], in older **(A,B)** and younger **(C**,**D)** populations. Shatavari ___ and Placebo – – –. Data are presented as mean ± standard deviation. *Significant main effect of supplement, *p* < 0.05.

In the older population, there was a significant main effect of supplementation on the number of LP repetitions completed per week across the training period (*F*(1,18) = 4.438, *p* = 0.049, η_*p*_^2^ = 0.198, intercept ≤ 0.001, covariate ≤ 0.001; Figure 2A); there was no significant effect of shatavari supplementation on the number of KE repetitions completed (*F*(1,19) = 1.259, *p* = 0.286, η_*p*_^2^ = 0.62, intercept ≤ 0.001, covariate ≤ 0.001; Figure 2B).

In the younger population, no main effect of supplementation was observed on repetitions completed per set for either LP (*F*(1,9) = 0.112, *p* = 0.745, η_*p*_^2^ = 0.012, intercept ≤ 0.001, covariate = 0.752, Figure 2C) or KE (*F*(1,9) = 2.030, *p* = 0.188, η_*p*_^2^ = 0.184, intercept = 0.01, covariate = 0.018, Figure 2D).

**FIGURE 3:**
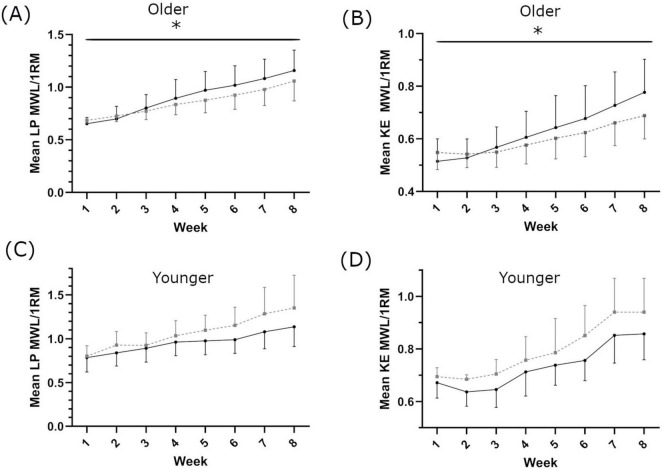
Maximum weight lifted each week for leg press [LP; **(A,C)**] and knee extension [KE; **(B**,**D)**], in older **(A,B)** and younger **(C**,**D)** populations. Shatavari ___ and Placebo – – –. Data are presented as mean ± standard deviation. *Significant main effect of supplement, *p* < 0.05. MWL; maximum weight lifted. 1RM, 1 repetition maximum.

**FIGURE 4:**
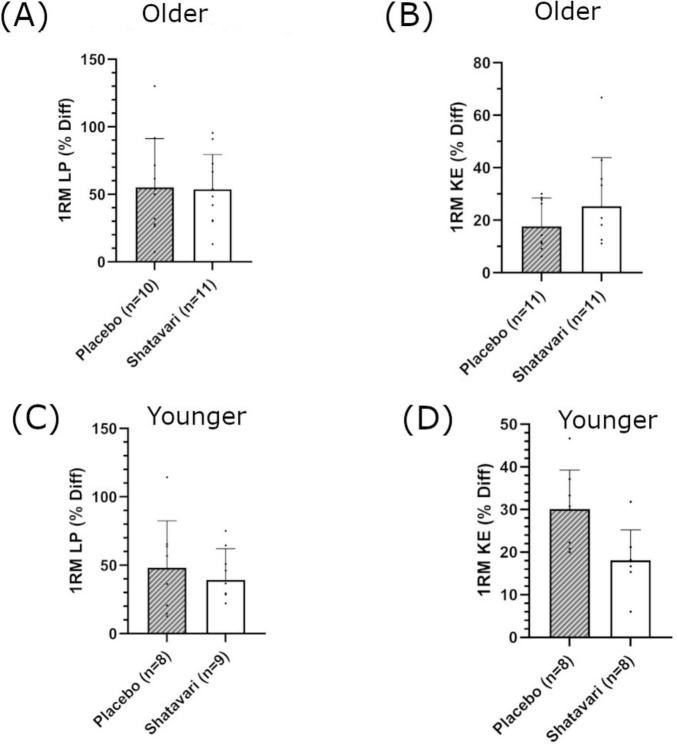
Percentage change in leg strength following 8 weeks of lower body resistance training. Leg press [LP; **(A,C)**] and knee extension [KE; **(B**,**D)**], in older **(A,B)** and younger **(C**,**D)** populations. Shatavari ___ and Placebo – – –. Data are presented as mean ± standard deviation. *Significant main effect of supplement, *p* < 0.05. 1RM, 1 repetition maximum; Diff, difference.

In older women, there was a main effect of supplementation on 1RM-adjusted MWL each week (LP: *F*(1,19) = 11.951, *p* = 0.03, η_*p*_^2^ = 0.386, intercept ≤ 0.001, covariate ≤ 0.001; KE: *F*(1,19) = 4.98, *p* = 0.043, η_*p*_^2^ = 0.198, intercept = 0.025, covariate ≤ 0.001; Figures 3A, B). In the younger population, there was no main effect of supplementation on 1RM-adjusted MWL each week (LP: *F*(1,9) = 2.888, *p* = 0.123 ηp^2^ = 0.243, intercept 0.246, covariate = 0.002 and KE: *F*(1,9) = 0.277, *p* = 0.612 η_*p*_^2^ = 0.030, intercept 0.544, covariate 0.151 respectively, Figures 3C, D). Eight weeks of resistance training significantly increased leg strength in the older population (1RM LP in SH 53.8 ± 25.7%; PL 55.2 ± 36.0%, main effect of time *p* = 0.001; 1RM KE in SH 25.3 ± 18.3%; PL 17.6 ± 10.8%, main effect of time *p* = 0.001, Supplementary Table 2). There was no main effect of supplementation (LP *p* = 0.575, KE *p* = 0.293 Figures 4A, B). In the younger population, leg strength significantly increased following training (1RM LP in SH 39.2 ± 22.8%, PL 48.0 ± 34.4%, *p* < 0.001; 1RM KE in SH 17.7 ± 7.6%, PL 30.1 ± 9.1, *P* < 0.001, Supplementary Table 2). There was no main effect of supplementation (LP *p* = 0.969, KE *p* = 0.436 Figures 4C, D). Training significantly increased skeletal muscle fiber size in both older (SH 23.3 ± 26.2%; PL 14.1 ± 21.2%, main effect of time *p* = 0.009) and younger women (SH 10.4 ± 16.7%; PL 12.9 ± 11.6%, main effect of time *p* = 0.03). There was no main effect of supplementation on skeletal muscle fiber size in either cohort. There was no main effect of supplementation on HGS in older or younger women (Supplementary Table 2).

### 3.3 Neuromuscular function

Training increased maximum voluntary isometric knee extension force in both cohorts; There was no main effect of supplementation observed in either population (Table 1).

**TABLE 1:** *Vastus lateralis* (VL) neuromuscular function outcomes evaluated before and after 8-weeks of placebo or shatavari supplementation and lower limb resistance training.

	Old						Young					
	**Placebo**		**Shatavari**		**Significance level**		**Placebo**		**Shatavari**		**Significance level**	
	**Pre**	**Post**	**Pre**	**Post**	**Supplement**	**Training**	**Pre**	**Post**	**Pre**	**Post**	**Supplement**	**Training**
**MVC (N)**	304.8 ± 57.8	326.6 ± 53.4	351.9 ± 55.7	357.6 ± 40.2	0.545	<0.001*	387.2 ± 69.5	454.5 ± 91.9	433.7 ± 152.0	484.6 ± 156.9	0.466	<0.001
**Amplitude (mV)**												
Resting	2.4 ± 0.5	2.6 ± 1.0	2.7 ± 1.2	2.7 ± 1.0	0.585	0.001*	3.9 ± 1.2	5.0 ± 1.0	4.2 ± 1.1	4.2 ± 1.2	0.171	0.502
MVC	3.0 ± 0.7	3.4 ± 1.0	2.9 ± 1.1	3.1 ± 1.0	0.641	0.044*	4.0 ± 1.2	5.1 ± 1.4	4.6 ± 1.1	4.7 ± 1.8	0.422	0.237
Potentiated	2.6 ± 0.6	3.0 ± 1.1	2.9 ± 1.2	2.9 ± 1.0	0.883	0.001*	3.9 ± 1.3	4.9 ± 1.0	4.2 ± 1.0	4.5 ± 1.3	0.497	0.329
Area (mV*ms)												
Resting	21.3 ± 4.4	20.5 ± 5.8	21.8 ± 6.9	20.8 ± 3.9	0.984	0.003*	28.3 ± 6.4	34.8 ± 7.4	32.4 ± 9.3	29.2 ± 7.8	0.125	0.485
MVC	20.9 ± 4.1	20.6 ± 4.6	19.3 ± 5.5	19.7 ± 5.5	0.987	0.007*	26.8 ± 6.8	31.6 ± 9.7	25.7 ± 7.4	26.0 ± 7.0	0.206	0.063
Potentiated	22.0 ± 4.7	21.8 ± 5.5	22.6 ± 4.4	22.3 ± 4.4	0.709	0.035*	28.6 ± 7.3	34.5 ± 6.9	31.9 ± 8.5	30.5 ± 8.5	0.252	0.457
CT (ms) * 1000												
Resting	122.6 ± 11.1	122.9 ± 14.7	121.4 ± 14.2	129.9 ± 14.3	0.386	0.041*	120.9 ± 11.4	121.2 ± 12.0	116.3 ± 6.0	120.0 ± 7.8	0.436	<0.001*
MVC	49.2 ± 7.4	48.6 ± 10.1	47.7 ± 12.2	48.5 ± 11.3	0.805	0.001*	53.1 ± 8.9	48.6 ± 12.5	47.4 ± 9.0	49.2 ± 11.8	0.183	0.001*
Potentiated	102.3 ± 9.2	103.0 ± 8.5	105.3 ± 10.1	104.9 ± 2.8	0.645	0.151	107.2 ± 6.6	108.0 ± 6.6	105.5 ± 4.4	110.0 ± 6.5	0.235	0.010*
**HRT (ms)**												
Resting	159.4 ± 61.5	142.8 ± 48.2	139.8 ± 50.3	136.6 ± 47.3	0.838	0.004*	117.8 ± 29.0	118.3 ± 17.4	94.6 ± 16.4	100.0 ± 14.7	0.329	<0.001*
MVC	21.9 ± 6.4	23.4 ± 11.8	22.6 ± 9.4	21.6 ± 8.3	0.481	<0.001*	22.7 ± 6.7	20.8 ± 8.6	17.9 ± 3.1	22.1 ± 4.0	0.428	0.324
Potentiated	141.0 ± 48.2	139.8 ± 42.5	139.0 ± 43.8	120.9 ± 34.2	0.021*	<0.001*	87.7 ± 10.1	90.5 ± 9.0	84.0 ± 26.0	89.6 ± 22.4	0.772	<0.001*
**PT(N)**												
Resting	85.9 ± 22.2	92.0 ± 25.7	95.3 ± 21.3	88.20 ± 13.16	0.218	0.002*	113.6 ± 30.1	110.3 ± 30.6	132.0 ± 24.7	144.5 ± 27.9	0.006*	<0.001*
MVC	10.8 ± 4.8	12.2 ± 9.3	10.9 ± 9.1	10.6 ± 8.5	0.541	<0.001*	16.7 ± 13.4	9.9 ± 7.8	9.9 ± 5.6	16.2 ± 10.8	0.029*	0.019*
Potentiated	139.2 ± 29.7	144.8 ± 33.5	146.6 ± 24.5	150.6 ± 17.1	0.980	<0.001*	173.8 ± 39.6	167.8 ± 34.8	200.0 ± 38.5	214.9 ± 38.6	0.010*	<0.001*

Data for young (22.9 ± 5.1 yr) and older (62.7 ± 5.3 yr) female cohorts are presented. CT, contraction time; HRT, half-relaxation time; MVC, maximum voluntary contraction; MVC, maximal voluntary contraction; mV, millivolts; ms, milliseconds; N, newtons. Data reported as mean ± standard deviation (mean ± SD)

*Statistically significant difference between SH and PL (*p* < 0.05).

Resistance training and shatavari supplementation differentially affected excitability (Mwave amplitude) and contractility (HRT, CT and peak twitch amplitude) parameters in younger and older women. Training increased Mwave amplitude in resting and maximally contracting muscle in older women, but did not alter Mwave amplitude in younger women. Training significantly shortened HRT during evoked contractions in resting muscle, in maximally contracting muscle and during potentiated twitches in older women (Table 1). Whereas in younger women, training significantly lengthened HRT during evoked contractions in resting muscle, during potential twitches, but not in maximally contracting muscle (Table 1).

In both age cohorts, training significantly increased peak twitch amplitude during evoked contractions in resting muscle, in maximally contracting muscle and during potentiated twitches (Table 1). In older women, training lengthened CT during electrically evoked contractions applied to resting muscle and maximally contracting muscle but not potentiated twitches (Table 1). In younger women training lengthened CT during evoked contractions in resting muscle and during potentiated twitches, but was decreased in maximally contracting muscle (Table 1).

Shatavari supplementation shortened HRT during a potentiated twitch in the older cohort only (Post training change: SH −11.74 ± 11.93%, PL 0.42 ± 14.73%, main effect of supplementation *p* = 0.021; Table 1). Shatavari supplementation did not alter peak twitch amplitude in the older cohort, but peak twitch amplitude was increased in the younger cohort at rest (SH 9.6 ± 7.2%, PL –2.7 ± 7.4%; main effect of supplement *p* = 0.006), in maximally contracting muscle (SH 30.8 ± 120.4%, PL –55.0 ± 79.0%; main effect of supplement *p* = 0.029), and during potentiated twitches (SH 7.9 ± 7.6%, PL –2.8 ± 7.9%; main effect of supplement *p* = 0.01). Data are presented in Table 1. There was no main effect of shatavari supplementation on Mwave amplitude or CT in either group (Table 1).

### 3.4 Proteomics

No individual proteins were differentially expressed between supplementation groups following resistance training. Following resistance training, differential protein expression analyses indicated that shatavari significantly influenced several *VL* proteomic pathways; many of these analyses are corroborated by visualizations of Markov clusters (Supplementary Figure 1).

In older women, the expression of acute phase (FDR < 0.001, LogFC 0.15) and innate immune system (FDR = 0.002, LogFC 0.13) proteins were increased by shatavari supplementation following resistance training. Shatavari supplementation combined with resistance training reduced the expression of such proteins in younger women (hemostasis/acute phase proteins FDR = 0.002, LogFC = −0.001; FCGR phagocytosis proteins FDR = 0.003, LogFC −0.053; complement cascade FDR < 0.001, LogFC −0.21) (Table 2).

**TABLE 2:** Skeletal muscle proteomic themes differentially regulated by shatavari following 8 weeks resistance training.

	Shatavari + resistance training					
			**Young**		**Older**	
**Theme**	**Young**	**Old**	**Mean FDR**	**Mean logFC**	**Mean FDR**	**Mean logFC**
Striated muscle contraction			0.1670	−0.0621	0.0000	−0.4298
Hemostasis/acute phase proteins			0.0015	−0.0083	0.0001	0.1472
Innate immune system			0.3137	0.0417	0.0015	0.1269
*FCGR phagocytosis*			0.0033	−0.0534	0.0001	0.2255
*Complement cascade*			0.0000	−0.2095	0.0000	0.4741
rRNA processing			0.7629	0.1037	0.0034	−0.0568
Translation			0.1837	0.1267	0.0104	−0.0244
ECM organization			0.0153	−0.0039	0.5882	−0.0236
*Collagen formation*			0.9088	0.0744	0.0043	−0.2762
*Laminin interactions*			0.8134	0.0417	0.0000	−0.3896
ECM proteoglycans			0.2656	0.0127	0.0028	−0.2300
Mitochondrial protein import			0.6952	0.1241	0.0249	−0.0416
RNA Polymerase I Transcription			0.9807	0.1508	0.0400	−0.4139
DNA methylation			0.9898	0.2010	0.0004	−0.7501
Apoptotic cleavage of cell adhesion proteins			0.0026	−0.5768	0.4846	−0.0685
O_2_/CO_2_ exchange in erythrocytes			0.0153	−0.2149	0.0885	0.4500

Changes represent a between supplementation groups comparison of pre-post training changes in the skeletal muscle proteome. Blue = decreased expression, Red = increased expression, Gray = no significant change in expression, FDR, false discovery rate; logFC, log fold change; FCGR, Fcgamma receptor; rRNA, ribosomal ribonucleic acid; ECM, extracellular matrix; DNA, dexoyribonucleic acid; O2, oxygen; CO2, carbon dioxide.

In older women, proteins related to striated muscle contraction were substantially decreased by shatavari supplementation during resistance training (FDR ≤ 0.001, LogFC −0.43); there was no effect of shatavari supplementation on these pathways in younger women (FDR = 0.167, LogFC −0.062) (Table 2 and Supplementary Figure 1).

Shatavari supplementation combined with resistance training reduced the expression of proteins related to rRNA processing in older women (FDR = 0.003, LogFC −0.057), but not younger women (FDR = 0.763, LogFC = 0.104). Further, expression of proteins related to translation was decreased in shatavari-supplemented older women (FDR = 0.01, LogFC −0.024), with no significant change in younger women (FDR = 0.184, LogFC = 0.13).

Extracellular matrix (ECM) organization proteins were diminished by shatavari supplementation in younger (FDR = 0.0153, LogFC −0.004), but not older women (FDR = 0.588, LogFC −0.024). However, proteins in collagen formation ECM subpathways, laminin interaction pathways and ECM proteoglycan pathways were decreased following shatavari supplementation in older women (Table 2). There was a shatavari-induced decrease in proteins involved in mitochondrial protein import in older women (FDR = 0.025, LogFC −0.042), with no such change observed younger women (FDR = 0.695, LogFC 0.12).

RNA Polymerase I transcription proteins decreased following training in shatavari-supplemented older women (FDR = 0.040, LogFC −0.41), with no significant change in younger women (FDR = 0.981, LogFC 0.15). DNA methylation proteins showed a significant decrease in shatavari-supplemented older women (FDR < 0.001, LogFC −0.75), with no significant change in younger women (FDR = 0.100, LogFC 0.20). Apoptotic cleavage of cell adhesion proteins showed a substantial decrease in younger women following shatavari supplementation (FDR = 0.003, LogFC −0.58), with no significant change in older women (FDR = 0.485, LogFC −0.068). Finally, proteins related to O_2_/CO_2_ exchange were decreased in younger women supplementing with shatavari during resistance training (FDR = 0.015, LogFC −0.22), but not in older women (FDR = 0.089, LogFC 0.45).

## 4 Discussion

### 4.1 General discussion

Here, we have demonstrated that shatavari supplementation during 8 weeks of leg resistance training increases accumulation of LP training load in older, but not younger, women. This increased training load did not lead to a greater increase in leg muscle strength or an additional increase in skeletal muscle fiber size compared to placebo-supplemented controls. Muscle contractility was altered by shatavari supplementation with shortened HRT following a maximal muscle contraction in older women and increased peak twitch amplitude in younger women. *VL* proteomic analyses demonstrated divergent molecular responses to resistance training and shatavari supplementation in younger and older women. Following resistance training combined with shatavari supplementation, older skeletal muscle was enriched in proteins related to the innate immune system and in acute phase proteins compared to placebo-supplemented controls. Notably, expression of innate immune system proteins was diminished, rather than enriched in shatavari-supplemented younger skeletal muscle. Proteins related to striated muscle contraction, ECM, transcription and translation were diminished by shatavari supplementation. In general, these molecular changes were unique to older skeletal muscle.

Anders et al. previously demonstrated that shatavari supplementation in trained young men during 8 weeks of upper body strength training evoked an increase in the accumulation of training load and increased repetitions until failure at 70% of bench press 1RM (6). Here, we have demonstrated an accelerated accumulation of LP training load in older (but not younger) women supplemented with shatavari. We did not observe any effect of shatavari supplementation on KE training load. Notably, KE exercise was completed prior to LP during each training session. We suggest that this may have ‘pre-fatigued’ one of the muscle groups involved in subsequent LP exercise and that may have revealed an effect of shatavari on skeletal muscle fatigue resistance. However, this increased training load did not lead to greater strength increases in the older shatavari-supplemented cohort. Our participants – in contrast to those of Anders et al. – were not regularly resistance training when recruited. All participants became stronger with training (c. 45% improvement in LP across all groups). We suggest that a study of longer duration or a study of trained participants may detect an effect of this shatavari-enhanced training load on leg strength accrual in older women. Shatavari supplementation did not alter training load in our younger cohort. The untrained nature of our younger cohort may explain this discrepancy with the work of Anders et al. but this does not explain the conflict with our findings in the older cohort (6). It may be that for the younger cohort we were underpowered to detect any difference between the supplementation groups. The trial in younger adults commenced shortly following a return to participant-facing research activities in the midst of the COVID-19 pandemic. Recruitment to this trial was extremely challenging in this environment and despite efforts to extend data collection for several years, financial and staffing constraints necessitated its termination.

We consider the purported mechanisms of shatavari action to be one possible explanation for the discrepancies in the effects of shatavari on training load between our younger and older cohorts and the young male cohort from Anders et al. (6). First, postmenopausal skeletal muscle is likely to be uniquely susceptible to one of the hypothesized mechanism of shatavari action. Shatavari contains phytoestrogens that bind to the estradiol receptor Busayapongchai and Siri (3); Sharma and Jaitak (4). Estradiol improves myosin binding function and muscle force production (9, 27, 28). Women experience an accelerated loss of muscle strength following the menopause, a phenomenon that hormone replacement therapy can ameliorate (29). We have previously shown that shatavari increases myosin regulatory light chain phosphorylation in the skeletal muscle of older women (7); this is a molecular indicator of improved skeletal muscle contractile function. The changes in training load discrepancy observed between our younger and older cohorts may be explained in part by this mechanism of shatavari action. Second, Anders et al. suggested that the exercise-induced oxidative stress during training sessions may have been mitigated by the antioxidant properties of shatavari (6). This remains plausible. During exercise or contractile activity, ROS are produced (30). These ROS can interact with cellular components, potentially impairing muscle function and growth, for example, myofibrillar calcium dynamics (14, 16, 31). Dietary supplements with antioxidant actions (e.g., polyphenol—rich fruit supplements, N-Acetylcysteine) have been speculated to counteract the adverse impacts of ROS on skeletal muscle function (14). Evidence for this mechanism of action remains speculative despite the demonstrable functional effects of such supplements (32–34). This potential mechanism of action is likely to be applicable to skeletal muscle regardless of age, sex or menopausal status. However, older skeletal muscle has been suggested to exist in a state of increased oxidative stress; skeletal muscle mitochondrial protein carbonylation is increased in older men and is correlated with muscle strength (35). It is therefore plausible that older adults could derive greater skeletal muscle benefit from a supplement with an antioxidant mechanism of action. It is notable that younger women are less prone to skeletal muscle fatigue and recover faster than their male counterparts (36–38). Higher levels of baseline fatigue-resistance in younger women may diminish the effects of shatavari and may explain the discrepancy between our findings and those of Anders et al. in younger men (6). Further, shatavari’s potential to accelerate functional recovery from exercise-induced muscle damage over a period of several days could help explain our observations of enhanced training load accrual in older women. Similar polyphenol-rich bioactives, such as tart cherry extract, have been shown to accelerate muscle recovery (17–19). We did not directly assess recovery markers in this study. However, if shatavari supports such recovery, this may have contributed to the observed improvements in serial performance over the course of twice-weekly training to muscle failure. This should be explored further in studies of shatavari supplementation under conditions of profound exercise-induced muscle damage.

We comprehensively characterized NMF in our participants. Shatavari supplementation of older women during 8 weeks leg resistance training significantly shortened HRT of the *VL* immediately following a maximal contraction. Rapid reloading of Ca^2+^ into the sarcoplasmic reticulum is required for subsequent muscle contraction. Estradiol deficiency and ROS have been suggested to inhibit sarcoplasmic reticulum Ca^2+–^ATPase (SERCA), thus lengthening HRT (39–41). Therefore, it is plausible that both estradiol-like and antioxidant mechanisms may contribute to the fatigue-resistance that we observed in the older shatavari-supplemented group. In the younger group, shatavari did not alter HRT. Higher circulating concentrations of estradiol, coupled with the lower background ROS burden in young skeletal muscle may prevent any effect of shatavari on baseline SERCA activity. However, PT was increased by shatavari supplementation in younger women. Estradiol increases muscle contractility in experimental animals and – as previously discussed – has been linked to enhanced muscle contractility in older humans (9). However, evidence of a relationship between circulating, physiologically normal estradiol concentrations and skeletal muscle contractility in younger women has not been convincingly established (42–44). This, coupled with our observation of no change in PT in shatavari-supplemented older women suggests that an estradiol-mediated mechanism is unlikely to be the sole driver of NMF changes in shatavari supplemented individuals. Indeed, the findings of Anders et al. in men support this notion. Older women, by virtue of their age and sex appear uniquely placed to benefit from both purported mechanisms of shatavari action. This requires further exploration.

We describe the effect of shatavari supplementation during resistance training on the *VL* proteome. The purpose of these analyses in the context of this study was to gain additional molecular insights into the mechanisms of shatavari action via comparison with a placebo supplemented group. We have analyzed these data for young and older participants separately. Comparisons between the age cohorts are qualitative, not statistical. To do this comprehensively constitutes a different study and is outside the scope of this manuscript. It is evident that the young and older cohorts have different molecular responses to supplementation with shatavari during a resistance training programme. Proteins related to striated muscle contraction, protein transcription and translation were diminished by shatavari supplementation following resistance training in older women. These changes were not observed in younger women. These observations are broadly in keeping with our previous work, in which we described a shatavari-induced decrease in *VL* proteins related to striated muscle contraction and translation in postmenopausal skeletal muscle following 6 weeks of shatavari supplementation alone i.e., in the absence of a resistance training intervention (7). We have previously speculated that a shatavari-induced decrease in proteins in pathways related to muscle contraction may represent a homeostatic downregulation of these proteins/pathways in response to an improvement in muscle contractility. Although proteins related to transcription and translation were diminished by shatavari supplementation following resistance training in older women, the skeletal muscle fiber hypertrophic response was unaffected. There was no difference in the degree of fiber hypertrophy between the placebo and shatavari supplementation groups. The mean increase in fiber cross-sectional area in the older shatavari cohort was higher (23% vs 14% in placebo group) than might be expected in older skeletal muscle where CSA evaluations were not subdivided by fiber type (this is a limitation of our study) (45). Despite our observation that there was no effect of supplement on fiber hypertrophy, we suggest that future work use our data to guide sample size calculations and ensure that this outcome measure is more completely evaluated.

ECM proteins were diminished in both older and younger participants supplementing with shatavari during resistance training. Notably, proteins related to laminin interactions and collagen formation were diminished in shatavari-supplemented older skeletal muscle. The ECM is now recognized for its crucial role in lateral force transmission, in dictating muscle shortening behavior, in effectively transmitting mechanical signals to the cell’s interior, facilitating downstream adaptations, including those of regenerative satellite cells, mechanical forces (46, 47). The diminished expression of ECM proteins following shatavari supplementation is interesting in the context of our observations of shatavari-induced changes in HRT and PT. When muscle fibers shorten, they must expand to maintain a constant volume (48). Constraining this radial expansion impedes muscle shortening (46, 49, 50). Therefore, remodeling of intramuscular connective tissue can alter myofibre contractility. Intramuscular connective tissue deposition is increased in older age. Older skeletal muscle displays a loss of collagen organization, an increase in total collagen and an increase in the elastic modulus of the ECM (51, 52). We suggest that a shatavari-induced decrease in ECM proteins may indicate ECM remodeling that favors effective contractile function. It is notable that no evidence exists regarding the effects of long-term exercise training on human skeletal muscle ECM remodeling. Acute bouts of resistance exercise appear to initially induce ECM catabolism, with a subsequent anabolic response. This biphasic response may be dysregulated in older adults (46).

Following resistance training combined with shatavari supplementation, older skeletal muscle was enriched in proteins related to the innate immune system. The converse was true in shatavari-supplemented younger skeletal muscle. Generally, lifelong physical activity is thought to ameliorate both the age-related increase in skeletal muscle background inflammation and the inflammatory response to a single exercise bout, thus leading to better muscle health (53, 54). Here, shatavari supplementation evoked what appears to be a contradictory effect. Proteinsrelated to FCGR (Fcγ receptor) phagocytosis were increased by shatavari in older skeletal muscle. FCGR is a well-established mediator of macrophage phagocytosis. A recent study has shown that resistance training-induced increases in skeletal muscle macrophages in older adults are positively correlated with muscle fiber hypertrophy (55). This was unexpected, as in that study, baseline CD11b + /CD206- macrophages were negatively associated with growth. Taken alongside our findings, this challenges the idea that recapturing a younger phenotype via promotion an ‘anti-inflammatory’ environment is key to fostering positive adaptations in older skeletal muscle. The mechanism by which shatavari might induce these effects in older, but not younger, skeletal muscle remains unclear. Our finding is particularly surprising given the well-known anti-inflammatory properties of its polyphenol constituents (56). It is possible that the ‘pro-inflammatory/adaptive’ effects of shatavari on aged skeletal muscle are indirect. These effects may be secondary to its impact on other aspects of myofibre function. For example, changes in ROS or extracellular matrix ECM proteins previously discussed could influence muscle function and training load with secondary effects on the skeletal muscle inflammatory environment (55). Despite no effect of shatavari on *VL* fiber hypertrophy being observed in this study, we suggest that future studies consider the effect of shatavari on skeletal muscle hypertrophy as a primary outcome. The present study can inform study design including sample size and explore relationships between skeletal muscle fiber size and macrophage infiltration in shatavari-supplemented individuals undergoing resistance training.

### 4.2 Limitations

This study has several important limitations. Participants cohorts were well-matched for BMI and physical activity inclusion criteria. However, baseline muscle mass and aerobic fitness were not assessed using gold-standard methods. Thus, any assumption that differences in muscle mass or fitness *per se* explain the differential effects of shatavari between cohorts would be unwarranted. Tests of NMF were conducted immediately before *VL* biopsy. Therefore, the skeletal muscle proteome that we have characterized depicts the proteome that exists following a bout of moderate resistance training. While mRNA expression might be altered over such a timeframe, it is unlikely that translational changes will have occurred. Proteomic analysis of *VL* samples was limited to 16/22 older participants and 12/17 younger participants. In the case of the older participants, this subgroup analysis was planned. Hypothesis-free pathway level (vs individual protein) analyses are not readily conducive to traditional sample size calculations. Published and unpublished data from our group suggested that we would detect meaningful changes in the tissue-level (skeletal muscle) proteome with 8 participants per group. The sample size for our studies was originally based on the best available data in the literature: 1RM bench press data presented by Anders et al. (6). It was determined that we required 16 participants per group to detect a similarly large change in knee extension KE 1RM (*d* = 1.03, α = 0.05, 1-β = 0.08). However, having recruited 11 participants per group, and having conducted a planned blinded interim analysis, it became evident that any real effect of supplementation on the primary outcome (KE 1RM) in this population was small in magnitude and we calculated using our KE means and SDs that we would require a sample size of 144 to detect an effect size of 0.47. For this reason, we considered it unethical to ask additional participants to undergo invasive procedures as part of this trial, particularly as sufficient skeletal muscle samples had been collected to gain pathway-level proteomic insights.

In our proteomic analysis, we employed the CAMERA Reactome algorithm. Alternatives are available, notably the PADOG algorithm which downweighs proteins that are found in multiple pathways. It has been our experience that PADOG’s downweighing algorithm particularly limits significant observations being described in immune pathways. It has been suggested that CAMERA results hold a lower degree of confidence than some other methods; however the FDRs calculated here suggest that any significant observations are not due to chance (57). Additionally, these data are corroborated by the Markov clusters generated by our STRING analyses; we consider these to be an important corroboration of the CAMERA results.

## 5 Conclusion

We have demonstrated that shatavari supplementation promotes the accumulation of greater LP training load in older – but not younger – women undergoing resistance training. This phenomenon was accompanied by evidence of increased contractility in older (shortened *VL* HRT following a maximal muscle contraction) and younger women, (increased peak twitch amplitude). These novel observations strongly support the notion that shatavari supplementation promotes neuromuscular fatigue resistance. Our results suggest that shatavari may evoke such effects via a number of mechanisms; all of these require further confirmation. First, an estradiol-like mechanism of action may exist wherein myosin binding function and muscle force production are enhanced by shatavari phytoestrogens. Second, exercise-induced oxidative stress may have been mitigated by the antioxidant properties of shatavari, thus mitigating the detrimental effects of ROS on skeletal muscle contractile function. Notably, estradiol deficiency and ROS have been suggested to inhibit SERCA, which can in turn lengthen HRT; shatavari supplementation may mitigate this. Finally, the *VL* expression of ECM proteins was diminished following shatavari supplementation in both age cohorts. Such changes could alter myofibre contractility. It is notable that older adults – and in particular older women – can be expected derive the greatest muscular benefit from shatavari supplementation if these proposed mechanisms are confirmed.

## Data Availability

The raw proteomics data presented in the study are deposited in PRIDE at https://www.ebi.ac.uk/pride/archive/projects/PXD056507/. Physiological data and proteomics outputs from analyses are deposited at: https://zenodo.org/records/14441162.

## Footnotes

1
http://seniam.org/sensor_location.htm

2
https://reactome.org/
