# Supplementary material for: Shatavari supplementation during eight weeks of resistance training increases training load, enhances skeletal muscle contractility and alters the skeletal muscle proteome in older women
Source: Front Nutr. 2025 Jan 6;11:1498674. doi: 10.3389/fnut.2024.1498674 (PMC11743497; doi:10.3389/fnut.2024.1498674)
Supplement: Supplementary file 1 [file Table_1.DOCX]

**Supplementary material for**

Title: **Shatavari Supplementation During Eight Weeks of Resistance Training Increases Training Load, Enhances Skeletal Muscle Contractility and Alters the Skeletal Muscle Proteome in Older Women.**

Running title: **Shatavari and resistance training**

Elsa Greed^1,2†^, Jack Pritchard^1†^, Lauren Struszczak^1^, Esra Bozbaş^1^, Georgia Ek^1^, Jordan Acheson^1,3^, Ben Winney^1^, Aaliyah Qadir^1^, Karl Ka-Lam Wong^1^, Joanna Bowtell^1^, Mary O’Leary^1*^

^1^ Faculty of Health and Life Sciences, Department of Public Health and Sport Sciences, University of Exeter, Exeter, United Kingdom.

^2^ School of Sport, Exercise and Rehabilitation Sciences, University of Birmingham, Edgbaston, Birmingham, UK.

^3^Department of Sport and Exercise Sciences, Institute of Sport, Faculty of Science and Engineering, Manchester Metropolitan University, Manchester, M1 5GD, UK

^†^These authors share first authorship.

**Supplementary table 1**. Medication,supplements, and contraception reported by participants.

| **Medication** | **No. participants reported taking (old)** | **No. participants reported taking (young)** | **Total** |
| --- | --- | --- | --- |
| Duloxetine | 1 | 0 | 1 |
| Octasa | 1 | 0 | 1 |
| Statins | 4 | 0 | 4 |
| Thyroxotine | 2 | 0 | 2 |
| Lansoprazole | 1 | 0 | 1 |
| Fluoxetine | 0 | 1 | 1 |
| Steroid inhaler | 0 | 1 | 1 |
| Co-dydramol | 0 | 1 | 1 |
| **Supplements** |  |  |  |
| Vitamin D | 13 | 1 | 14 |
| Vitamin C | 2 | 1 | 3 |
| Vitamin B | 1 | 0 | 1 |
| Tumeric | 4 | 0 | 4 |
| Magnesium | 4 | 0 | 4 |
| Cod liver oil | 4 | 0 | 4 |
| Evening primrose oil | 3 | 0 | 3 |
| Multivitamins | 9 | 4 | 13 |
| Collagen | 2 | 0 | 2 |
| Calcium | 3 | 0 | 3 |
| Biotin | 1 | 0 | 1 |
| Omega-3 | 1 | 0 | 1 |
| Zinc | 1 | 0 | 1 |
| Curcumin | 1 | 0 | 1 |
| Lions Maine | 1 | 0 | 1 |
| Ashwaghanda | 1 | 0 | 1 |
| Glucosamine | 1 | 0 | 1 |
| Iron | 0 | 1 | 1 |
| **Contraception** |  |  |  |
| Copper coil | n/a | 1 | 1 |
| Implanon implant | n/a | 1 | 1 |
| Rigevidon | n/a | 2 | 2 |
| Noriday | n/a | 1 | 1 |
| Nexplanon | n/a | 1 | 1 |

**Supplementary table 2**. 1RM knee extension, leg press and hand grip strength pre- and post-training in older and younger women in placebo and supplementation groups.

|  | **Older Women** | | | | | **Younger Women** | | | | |
| --- | --- | --- | --- | --- | --- | --- | --- | --- | --- | --- |
|  | **Shatavari** | | **Placebo** | | **Shatavari** | | | **Placebo** | |  |
|  | *Pre* | *Post* | *Pre* | *Post* | *Pre* | | *Post* | *Pre* | *Post* |  |
| **KE 1RM (Kg)** | 59.8 ± 3.5 | 73.8 ± 12.3 | 59.8 ± 9.2 | 70.3 ± 12.5 | 93.6 ± 15.9 | | 109.8 ± 14.1 | 70.4 ± 12.2 | 91.2 ± 14.2 |  |
| **LP 1RM (Kg)** | 138.7 ± 14.0 | 206.3 ± 42.1 | 121.0 ± 29.5 | 180.2 ± 47.5 | 154.6 ± 59.3 | | 221.1 ± 96.5 | 116.2 ± 64.4 | 168.3 ± 84.7 |  |
| **HGS (Kg)** | 22.9 ± 3.8 | 24.1 ± 4.6 | 25.9 ± 4.4 | 26.9 ± 4.8 | 27.6 ± 5.2 | | 27 ± 3.6 | 23 ± 1.5 | 25.5 ± 2.5 |  |

*KE; knee extension. 1RM; 1 repetition maximum. Kg; kilograms. LP; leg press. HGS; handgrip strength.*


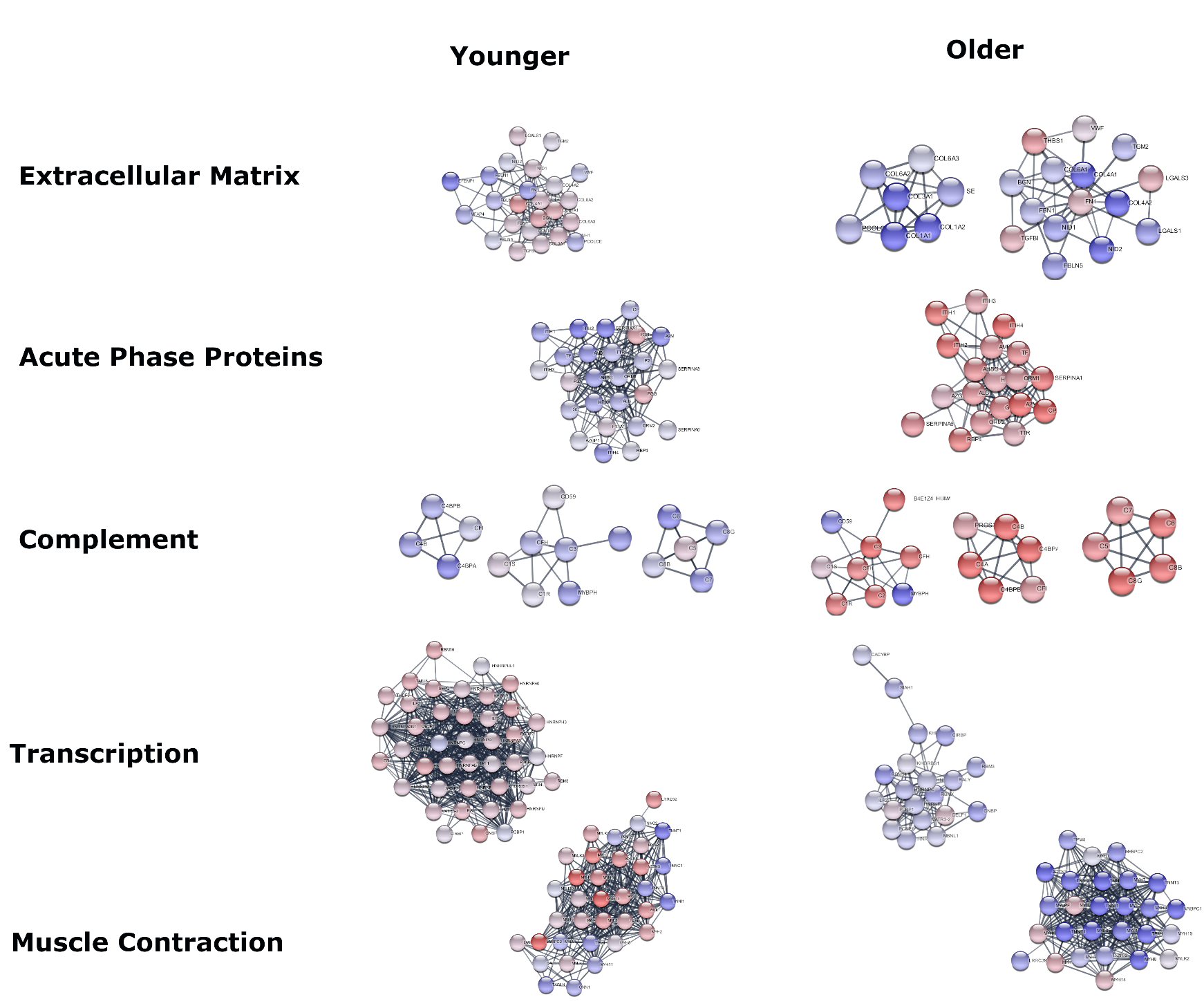


**Supplementary Figure 1.** Visualisation of skeletal muscle protein networks most prominently altered by shatavari supplementation during eight weeks resistance training. Log2 fold changes of skeletal muscle protein expression between placebo and shatavari supplemented female participants were mapped to nodes using a blue−white−red gradient. To aid readability, protein names are indicated by their official gene symbol. **Note: FASEB allow free format initial submissions. If invited to re-submit following review, this supplementary figure will be uploaded as a separate high resolution image which allows node names to be better explored.**
